# Hydroxyethyl starch 6%, 130/0.4 vs. a balanced crystalloid solution in cardiopulmonary bypass priming: a randomized, prospective study

**DOI:** 10.1186/1749-8090-8-71

**Published:** 2013-04-08

**Authors:** Hasan Alper Gurbuz, Ahmet Baris Durukan, Nevriye Salman, Murat Tavlasoglu, Elif Durukan, Halil İbrahim Ucar, Cem Yorgancioglu

**Affiliations:** 1 Department of Cardiovascular Surgery, Medicana International Ankara Hospital, Ankara, Turkey; 2 Department of Biology, Hacettepe University Faculty of Science, Ankara, Turkey; 3 Department of Anesthesiology, Medicana International Ankara Hospital, Ankara, Turkey; 4 Department of Cardiovascular Surgery, Diyarbakir Military Hospital, Diyarbakir, Turkey; 5 Department of Public Health, Ankara Baskent University, Ankara, Turkey

**Keywords:** Hetastarch, Coronary artery bypass, Outcome assessment

## Abstract

**Background:**

Since the advent of cardiopulmonary bypass, many efforts have been made to avoid the complications related with it. Any component of the pump participates in occurrence of these adverse events, one of which is the type of prime solution. In this study, we aimed to compare the effects of 6% hydroxyethyl starch 130/0.4 with a commonly used balanced electrolyte solution on postoperative outcomes following coronary bypass surgery.

**Methods:**

Two hundred patients undergoing elective coronary bypass surgery were prospectively studied. The patients were randomized in to two groups. First group received a balanced electrolyte solution and the second group received 6% hydoxyethyl starch 130/0.4 as prime solution. The postoperative outcomes of the patients were studied.

**Results:**

The mean age of the patients was 61.81 ± 10.12 in the crystalloid group whereas 61.52 ± 9.29 in the HES group. There were 77 male patients in crystalloid group and 74 in HES group. 6% hydroxyethyl starch 130/0.4 did not have any detrimental effects on renal and pulmonary functions. The intensive care unit stay and postoperative hospital length of stay were shorter in hydroxyethyl starch group (p < 0.05 for each). Hydroxyethyl starch did not increase postoperative blood loss, amount of blood and fresh frozen plasma used, but it decreased platelet concentrate requirement. It did not have any effect on occurrence of post-coronary bypass atrial fibrillation (p > 0.05).

**Conclusions:**

6% hydroxyethyl starch 130/0.4 when used as a prime solution did not adversely affect postoperative outcomes including renal functions and postoperative blood transfusion following coronary bypass surgery.

## Background

Increased number of patients are undergoing coronary artery bypass grafting (CABG) and efforts to prevent adverse events and improve outcomes are done. One of the most important problems following cardiac surgery is renal failure. Use of cardiopulmonary bypass (CPB), blood and constituents, various drugs and infusion of large volumes of fluids influence the renal functions. Occurrence of perioperative renal impairment causes increased mortality and morbidity [1]. Modifications on every component of CPB are studied to decrease the risk of renal failure particularly related with CPB use.

Cardiopulmonary bypass priming solution and volume are of special importance since it directly affects renal functions. Hemodilution due priming volume, continuous flow pattern of CPB, use of various drugs during CPB and occurrence of systemic inflammatory response syndrome (SIRS) adversely affect renal functions [2].

Hydroxyethyl starch (HES) is commonly used in current practice as a volume expander in trauma, shock, cardiac and other major surgeries and vast numbers of reports are being published with conflicting results. Still there is no consensus on the renal effects of HES solutions.

In this randomized prospective study, we aimed to document the effects of 6% hydroxyethyl starch 130/0.4 on postoperative outcomes and occurrence of atrial fibrillation (AF) in patients undergoing on-pump CABG surgery.

## Methods

A prospective and randomized study has been carried out. The study was approved by the “Medicana International Ankara Hospital Ethics Committee” and written informed consent was taken from every patient. The only inclusion criteria were isolated on-pump CABG procedure. Both genders were accepted. There were no age or weight restrictions. Exclusion criteria were; repeat cardiac surgery, emergent surgery, preoperative coagulation disorder, preoperative clopidogrel use, preoperative congestive heart failure, preoperative renal dysfunction (serum creatinine > 1.3 mg/dl), preoperative hepatic dysfunction (serum aspartate/alanine amino transferase > 40U/l), preoperative electrolyte imbalance, history of pancreatitis and known hypersensitivity to HES. Between October 2011 and April 2012, after the inclusion and exclusion criteria were employed 200 isolated CABG cases were studied. The patients were then randomized as follows: each patient was given a number according to chronological order beginning from 1. The odd numbered patients (n:100) were administered 6% HES 130/0.4 in 0.9% sodium chloride (Voluven® %, Fresenius Kabi, Bad Homburg, Germany) as CPB prime solution and even numbered patients (n:100) were administered a balanced multielectrolyte solution (Isolyte-M®, Eczacıbaşı-Baxter, İstanbul: dextrose monohydrate, 40 mEq/l sodium, 40 mEq/l chloride, 35 mEq/l potassium, 15 mEq/l phosphate, 20 mEq/l acetate; 400 mOsm/l, 170 kCal/l).

Preoperative acetylsalicylic acid 100 mg/day was continued in all patients prior to the day of surgery. All patients were premedicated with 10 mg of oral diazepam. Anesthesia was induced with etomidate 2 mg/kg, fentanyl 1 μg/kg, vecuronium 1 mg/kg; isofluorane 1 MAC was used for anesthesia maintenance. Intraoperative arterial and central venous pressure monitorization were done.

The cardiopulmonary bypass (CPB) circuit was primed with 1,500 ml of determined solution. In both groups 5,000 units of heparin was added. After anticoagulation with heparin (300 U/kg), activated clotting time (ACT) was kept over 400 seconds. CPB was established using a roller pump with a membrane oxygenator (Dideco Compactflo Evo, Sorin Group, Mirandola Modena, Italy). The average flow rate varied from 2.3 to 2.4 l/min/m^2^. Surgery was performed under mild hypothermia (33°C). Mean arterial pressure was kept between 45 to 70 mm Hg. All patients were rewarmed to 37°C (nasopharyngeal temperature) before weaning from CPB. Heparin was neutralized with 1:1 protamine sulfate.

Cold (4-8°C) blood cardioplegia of 1000 ml (25 mEq/l potassium) was administered after aortic cross clamping, and 500 ml repeat doses were given every 15 to 20 minutes (antegrade and from venous bypass grafts, retrograde in patients with left main coronary disease). Terminal warm blood cardioplegia (36-37°C) was given prior to aortic clamp release.

The operation room temperature was kept at 20-21°C.

In postoperative period rate of fluid infusions were adjusted according to hemodynamic measurements. Central venous pressure was maintained between 8–12 mm Hg.

Packed red blood cell was given if the hematocrit level fell below 25%. Fresh frozen plasma and platelet concentrates were administered in cases of documented postoperative coagulation abnormalities (international normalized ratio > 1.5, activated partial thromboplastin time > 60 s and platelet count < 80,000/mm^3^) or suspected postoperative platelet dysfunction and factor deficiency.

The decision for re-exploration for hemorrhage was made when 200 ml/hour of drainage was documented on two consecutive hours despite measures taken or more than 300 ml/hour drainage.

On postoperative day 1, all patients were administered metoprolol (50 mg/day) or carvedilol (3.125-6.25 mg/day) and N-acetylcysteine (oral: creatinine < 1.3 mg/dl; intravenous: creatinine > 1.3 mg/dl) and continued. All patients were routinely administered low molecular weight heparin in prophylactic dose.

Atrial fibrillation was diagnosed based on electrocardiogram. All patients were ECG monitored continuously during the intensive care unit (ICU) stay and for the first 24 hours in the ward. Soon ECG was immediately performed in cases of irregular pulse, palpitation or symptoms related with possible AF.

In cases with AF, if required intravenous metoprolol was administered for heart rate control. For rhythm control, intravenous amiodarone was administered as intravenous 300 mg loading dose in 1 hour, followed by 900 mg in 24 hours and followed by oral amiodarone 200 mg three times a day. In refractory cases 450 mg additional intravenous infusion was given in 12 hours period. If no response was noted after 48 hours, electrical cardioversion was employed. Low molecular weight heparin dosage was switched to therapeutic interval. In cases of permanent AF development, oral warfarin was administered.

Primary outcome variables included mean time to extubation, ICU and postoperative hospital length of stay, incidence of renal dysfunction (based on the finding that peak creatinine value was 1.5 or greater times the preoperative value), postoperative stroke, postoperative total amount of blood loss, postoperative exploration for hemorrhage, number of used blood and blood products and in hospital mortality.

### Statistical analysis

Statistical analyses were performed using SPSS software for Windows version 17.0 (Statistical Package for the Social Sciences Inc, Chicago, IL, USA). Continuous variables were expressed as ‘mean values ± standard deviation (SD)’. Categorical variables were expressed as number and percentages. Demographic characteristics and outcomes of the groups were compared using “independent samples *t*-test” for continuous variables, and, ‘chi-square test’ and ‘Fisher’s exact test’ for categorical variables. Statistical significance was set as ‘p < 0.05’.

## Results

The mean age of the patients was 61.81 ± 10.12 in the crystalloid group whereas 61.52 ± 9.29 in the HES group (p > 0.05). There were 77 male patients in crystalloid group and 74 in HES group (p > 0.05). Preoperative demographic findings and intraoperative characteristics of the patients are given in Table 1.

**Table 1:** Comparison of the two groups by preoperative and intraoperative characteristics

**Factor**	**Isolyte-M® group (n:100)**	**Voluven® 6% group (n:100)**	p value*
	**Mean ± SD**	**Mean ± SD**	
Age	61.81 ± 10.12	61.52 ± 9.29	0.833
BMI (kg/m^2^)	27.88 ± 3.96	29.02 ± 4.61	0.063
LVEF (%)	53.72 ± 10.81	52.33 ± 11.08	0.370
Cross-clamp time (min)	53.57 ± 20.12	55.58 ± 17.22	0.449
CPB time (min)	79.69 ± 27.93	82.57 ± 23.98	0.435
Graft #	3.22 ± 1.06	3.10 ± 0.90	0.390
	n:%	n:%	p value**
Patient Total	100	100	
Male sex	77	74	0.622
Current/Ex-smoker	67	63	0.553
Diabetes Mellitus	46	42	0.569
Hypertension	65	62	0.659
Dyslipidemia	79	76	0.611
Preoperative β-blocker use	40	47	0.318
Peripheral Arterial Disease^a^	1	6	0.118***
Stroke	-	1	1.000***
Carotid Disease^b^	7	5	0.552***
COPD/Asthma	14	15	0.841

*independent samples *t*-test.

**chi-square test.

***Fisher’s exact test.

^a^History of therapeutic vascular intervention, history of claudication, angiography/non-invasive proven peripheral arterial disease.

^b^History of carotid intervention or angiographic/non-invasive proven >40% stenosis of either carotid.

BMI: body mass index, LVEF: left ventricular ejection fraction.

CPB: cardiopulmonary bypass, COPD: chronic obstructive pulmonary disease.

There was not a statistically significant difference when intubation times, postoperative drainage, amount of red blood cell and fresh frozen plasma used and postoperative adverse events (renal failure, stroke, mortality) were compared. Number of platelet concentrate used was lower in the HES group (p < 0.05). Postoperative ICU and postoperative hospital length of stay were shorter in HES group (p < 0.05) (Table 2). No mortality was noted during the study period.

**Table 2:** Comparison of the two groups by postoperative variables

	**Isolyte-M® group**	**Voluven® 6% group**	
	**Mean ± SD**	**Mean ± SD**	p value*
ICU intubation time, hours	10.38 ± 9.04	9.38 ± 2.64	0.290
Length Of Stay			
ICU, hours	47.93 ± 12.01	45.25 ± 5.86	0.046
Postoperative, days	6.14 ± 2.55	5.47 ± 1.20	0.019
Drainage tubes removed, hours	36.36 ± 10.39	36.12 ± 13.32	0.886
Total amount of drainage, ml	741.75 ± 448.58	680.30 ± 332.92	0.273
Number of FFP used	1.05 ± 1.32	1.02 ± 1.40	0.877
Number of packed RBC used	1.82 ± 1.65	1.63 ± 1.50	0.397
Number of PC used	0.61 ± 1.92	0.15 ± 0.98	0.035
	n:%	n:%	p value**
Postoperative exploration for hemorrhage	2	5	0.445
Postoperative AF	15	19	0.451***
Renal Dysfunction^a^	6	9	0.421
Postoperative Stroke	1	1	1.000

*Independent samples *t*-test.

** Fisher’s exact test.

***chi-square test.

^a^defined when peak creatinine value was 1.5 or greater times the preoperative value.

ICU: intensive care unit, FFP: fresh frozen plasma.

RBC: red blood cell, PC: platelet concentrate.

AF: atrial fibrillation.

Atrial fibrillation incidence was 19% in HES group whereas 15% in crystalloid group (p > 0.05).

## Discussion

Coronary bypass surgery is the most frequently performed cardiac operation worldwide and modifications are made in order to maintain more reasonable results [3]. Various improvements are recorded on surgical techniques, CPB devices, drugs and perioperative management. Postoperative outcomes especially renal functions are very important in these patients due to the direct relation between mortality and morbidity. In this report, we also hypothesized to document improvements in perioperative management by using HES solutions as priming solution.

During CPB, derangements in multiple organ systems occur, and the resultant SIRS is the main reason for postoperative morbidity and mortality. The main causes of these changes are the contact with foreign surfaces, changes in coagulation and fibrinolytic systems, activation of the complement system, hemodilution and hypothermia. Also endotoxemia and ischemia-reperfusion injury have adverse effects. Any component of CPB system has direct influence on postoperative outcomes. Two of the most important components are the type and volume of priming solution [4,5].

Various types of priming solutions have been researched and employed, but today still there is no consensus on ideal priming solution in clinic use that can prevent SIRS, fluid retention and hypercoagulation. Hydroxyethyl starch is used very frequently as a priming solution in cardiovascular surgery and as a volume expanding agent in perioperative care and in trauma. Many reports were published concerning the results following HES use, but very recently some of them were retracted. This shaded the formed concept on HES solutions and created a doubtful era. Today, there is still a controversy on the effects of HES particularly on coagulation and renal functions.

Choi et al. [6] studied the effects of HES when used as a priming solution in comparison with human albumin and they did not find any difference on coagulation variables, postoperative blood loss, transfusion requirements and inflammatory response. In our study, we compared HES with a commonly used crystalloid solution and found similar results; HES did not cause detrimental results on postoperative outcomes.

Liou et al. [7] reported comparison between three different priming solutions; ringers lactate, human albumin and %10 HES. Time to extubation, ICU stay and hospital stay did not differ among the groups. The inflammatory cytokines TNF-α, IL-1β and IL-6 levels were also measured following CPB and no statistically significant difference was reported. There was statistically significant difference in postoperative body weight gain, HES and human albumin groups caused less weight gain compared to ringers lactate. This was because hypo-oncotic prime solutions lead to interstitial fluid expansion more than colloids. We also documented similar results, but we also studied postoperative outcomes. We did not study the inflammatory cytokines.

Kuitunen et al. [8] reported the effects of HES used for priming on coagulation and concluded that HES use increased blood loss. They revealed that less stable thrombi were formed documented with thromboelastography. They also concluded that HES in cardiac surgery may increase blood loss. In our study, we did not find any difference concerning the blood loss between the crystalloid and HES groups, HES did not adversely affect postoperative bleeding.

Tiryakioglu et al. [9] designed a similar study of prime solutions and compared ringers lactate with HES. They documented unfavorable effects of HES on renal functions, but the urea and creatinine levels were in the normal range in both groups. They found no statistical difference in amount of postoperative bleeding, time to extubation, intensive care unit stay and discharge times. They only noted statistically significant difference in net volume balance in favor of HES group. We did not document any adverse effect on renal functions.

Yap et al. [10] studied gelatin and HES as prime solutions and compared postoperative outcomes like intraocular pressure, blood profile and blood loss. They designated intraocular pressure as a marker to determine plasma oncotic pressure and they found that HES had had significant favorable results compared to gelatin. There was no difference concerning blood profile, blood loss and other postoperative outcomes.

In a meta-analysis, it was documented that use of HES did not cause any impairment in renal functions and also no difference was found considering the risk of complications, reoperation and mortality [11]. Very recently, Akkucuk et al. [12] documented their results on use of HES 130/0.4 on pediatric patients undergoing CPB and revealed no adverse effects on renal functions and other events.

In cardiac surgery, the effects of HES solutions also were studied for volume replacement in the postoperative period. The results of the studies were similar to those when HES solutions were used for priming. There were no adverse effects documented on postoperative coagulation parameters and postoperative renal functions [13,14].

The effects of HES on AF can be regarded as a special issue. Atrial fibrillation is the most common rhythm disturbance following cardiac surgery and many efforts are being made to reduce the risk of occurrence of AF. It was revealed that there was a strong correlation between the AF and inflammation [15-17]. The anti-inflammatory effects of HES solutions have been documented [18,19]. In our study, we also studied the effects of HES used as a prime solution on occurrence of postoperative AF. We did not note any difference between the groups.

## Conclusions

In this prospective randomized study, we did not document any difference between HES and crystalloid solutions used for CPB priming regarding postoperative outcomes like postoperative bleeding, renal functions and the use of blood and FFP. The number of used PC was less and the hospital length of stay and ICU stay were shorter in HES group. The results may differ in high risk patient groups and further studies with increased number of patients should be made particularly on patients with renal failure and coagulation disorders.

## Abbreviations

CABG: Coronary artery bypass grafting; CPB: Cardiopulmonary bypass; SIRS: Systemic inflammatory response syndrome; HES: Hydroxyethyl starch; AF: Atrial fibrillation; ICU: Intensive care unit

## Competing interests

The authors declare that they have no competing interests.

## Authors’ contributions

GHA: study concepts, study design, definition of intellectual content, literature research, data acquisition, data analysis, manuscript preparation, manuscript editing; DAB: study concepts, study design, definition of intellectual content, literature research, data acquisition, data analysis, manuscript preparation, manuscript editing SN: definition of intellectual content, literature research, data acquisition, manuscript preparation, manuscript editing; DE: data analysis, statistical analysis, manuscript editing; TM: definition of intellectual content, data acquisition, data analysis, manuscript editing; UHI: study concepts, study design, definition of intellectual content, data acquisition, data analysis, manuscript editing; YC: study concepts, study design, definition of intellectual content manuscript preparation, manuscript editing, supervision. All authors read and approved the final manuscript.
